# Blood meal sources and entomological inoculation rates of anophelines along a highland altitudinal transect in south-central Ethiopia

**DOI:** 10.1186/1475-2875-12-76

**Published:** 2013-02-23

**Authors:** Abebe Animut, Meshesha Balkew, Teshome Gebre-Michael, Bernt Lindtjørn

**Affiliations:** 1 Center for International Health, University of Bergen, Bergen, Norway; 2 Aklilu Lemma Institute of Pathobiology, Addis Ababa University, P. O. Box 1176, Piazza, Ethiopia

## Abstract

**Background:**

The role of anophelines in transmitting malaria depends on their distribution, preference to feed on humans and also their susceptibility to *Plasmodium* gametocytes, all of which are affected by local environmental conditions. Blood meal source and entomological inoculation rate of anophelines was assessed along a highland altitudinal transect in south- central Ethiopia.

**Methods:**

Monthly adult anopheline sampling was undertaken from July 2008 to June 2010 in Hobe (low altitude), Dirama (mid altitude) and Wurib (high altitude) villages located at average elevations of 1800 m, 2000 m and 2200 m, respectively. Anophelines were collected using CDC light trap, pyrethrum space spray catches (PSC) and artificial pit shelter methods. Upon collection, females were categorized according to their abdominal status and identified to species. Their human blood index, sporozoite rate and entomological inoculation rate was determined.

**Results:**

A total of 4,558 female anophelines of which *Anopheles arabiensis* was the most prevalent (53.3%) followed by *Anopheles demeilloni* (26.3%), *Anopheles christyi* (8.9%)*, Anopheles pharoensis* (7.9%) and *Anopheles cinereus* (3.6%) were caught and tested for blood meal source or sporozoite infection depending on their abdominal status. The proportions of human fed and bovine fed *An. arabiensis* were generally similar. In the low altitude village, there were 0.3% (1/300) and 0.2% (1/416) *Plasmodium falciparum* infected *An. arabiensis* among the CDC trap catches and PSC respectively. The percentage of *Plasmodium vivax* infected *An. arabiensis* were 3% (9/300) and 0.7(3/416) among the CDC and PSCs respectively in the village. In addition, there were 1.4% (1/71) and 50% (1/2) *P. vivax* infected *An. pharoensis* from the CDC light trap and PSCs, respectively. In the mid altitude village, 2.5% (1/40) and 1.7% (1/58) from among the CDC and PSCs of *An. arabiensis* respectively carried *P. vivax* sporozoites. Among the CDC light trap catches; there were 3.7 and 0 *P. falciparum* infective bites per year per household for *An. arabiensis* in the years July 2008 to June 2009 and July 2009 to June 2010 respectively in the low altitude village. The corresponding numbers for *P. vivax* infective bites for *An. arabiensis* were 33 and 14.5 in the same village. Space spray catches revealed 0.32 *P. vivax* infective bites per household for *An. pharoensis* during the first year in the low altitude village.

**Conclusion:**

*Anopheles arabiensis* was the most prevalent vector of *P. vivax* and *P. falciparum* malaria in the low and mid altitude villages followed by *An. pharoensis*. Annual entomological inoculation rates showed that vivax malaria transmission was higher than that of the falciparum and both decreased with increase in altitude.

## Background

*Plasmodium falciparum* and *Plasmodium vivax* are the most prevalent malaria parasites in Ethiopia [1] of which the first is the most notable cause of sickness and death. Transmission of the disease is unstable and occurs mainly from September to December following the June-September rains while the minor transmission occurs in April to May following the February-March small rains. Areas between 1,500 m and 2,500 m altitude have been affected by epidemics at intervals of 5–8 years while those below 1, 500 m are affected by seasonal transmission. Moreover, the increasing magnitude of the global temperature and ecological changes [2-5] might have contributed in the expansion of the disease to areas higher than 2, 500 m altitude [6-8].

*Anopheles arabiensis* is the principal malaria vector in Ethiopia [9] while *Anopheles pharoensis*, *Anopheles funestus* and *Anopheles nili* are secondary vectors [1,10,11]. *Anopheles arabiensis* is adapted to diverse ecology, feeding preference, seasonal occurrence and vectorial capacity resulting in diverse spatial and temporal malaria transmission patterns [12-14]. The role of anophelines in transmitting the disease depends on their occurrence and preference to feed on humans [15], which in turn is affected by local socio-economic as well as environmental factors [14,16]. Thus, preventing humans from the bite of vectors can reduce malaria transmission. However, implementation of prevention tools requires knowledge on occurrence, feeding behaviour and entomological inoculation rate of the vector in the local setting [17,18].

Preference of anophelines to feed on humans can be estimated using human blood index (HBI). HBI is the proportion of human fed among a total of fresh fed anophelines. However, as a vector may feed on alternative hosts depending on availability and accessibility, it remains imperative to assess its blood meal source in local settings. Tests such as enzyme-linked immunosorbent assay (ELISA) [19,20], precipitin test [10] and polymerase chain reaction (PCR) [16] can be employed to identify the blood meal source of a vector of which the first is preferable.

Risk of malaria infection can be measured using entomological inoculation rate (EIR) [20,21]. EIR of a vector depends on its human biting frequency and susceptibility to *Plasmodium* gametocytes [15,22]. It is the product of the human biting rate (HBR) and the sporozoite rate (SR) [17]. The human bait catch is considered as the gold standard method to determine HBR or human landing collection (HLC) [23]. However, it is technically difficult to replicate and unethical in areas where malaria parasites are resistant to drugs [24] and where other mosquito-borne diseases are common. Indoor spray collection and exit trap have been used in some cases but are less sensitive as the anophelines could be less directly associated with feeding on humans [16,24]. The Centers for Disease Control (CDC) light trap hang nearby sleeping people, at night, can also be used to estimate HBR as it catches mosquitoes that attempt to feed on humans [22,25,26]. However, the relationship between a CDC light trap catch and a HLC varies by locality based on the behaviour of the local vectors. SR is the proportion of vectors that carry *Plasmodium* sporozoites in their salivary glands. Anophelines can be diagnosed for sporozoite infection by dissecting their salivary glands [23], by polymerase chain reaction (PCR) [27] or by ELISA [19] of their thorax and head. In the present study, human blood indices and entomological inoculation rates of anophelines was assessed in a highland malarious area of south-central Ethiopia [28,29].

## Methods

### Study area

Adult anopheline sampling was undertaken along a highland transect of south-central Ethiopia consisting of Hobe (low altitude), Dirama (mid altitude) and Wurib (high altitude) villages once per month for 24 months (July 2008 to June 2010). The villages are located at average elevations of 1,800 m, 2,000 m and 2,200 m, respectively. The low altitude village (N=08^0^01^′^.912; E=038^0^29^′^.179) is adjacent to Odamo stream, the mid (N=08^0^10^′^.061; E=038^0^25^′^.142) to Akamuja stream and the high (N=08^0^04^′^.877; E=038^0^17^′^.991) to Assas stream and Beko Swamp. The streams serve as permanent anopheline breeding habitats during dry seasons [30]. The average annual rainfall of the area is 1,135 mm while the annual average minimum and maximum temperature is 11.5°C and 25°C respectively. The average number of occupants per house in the study villages is 4.3 and the inhabitants keep their small number of livestock in their residential houses during the night. Most of the houses were constructed of mud plastered wood and thatched roof. Like the rest of the country, malaria vector control is one of the strategies for the prevention and control of the disease and activities include implementation of LLINs (PermaNet®) in the low and mid altitude villages and a once per year indoor residual spraying in the low altitude village (personal communication with district health officers). During the study period, LLINs ownership was 28.5% (Woyessa, personal communication).

### Collection, identification and processing of anopheline mosquitoes

Anophelines were sampled using CDC light traps (John W. Hock Ltd, Gainesville, FL., USA) and pyrethroid space spray collections (PSCs) from indoors and artificial pit traps from outdoors [23]. In each village, CDC light traps were set running from 6:00 pm to 6:00 am for two consecutive nights in 10 houses (one trap/ house) and the same was repeated in another 10 houses resulting in 40 CDC trap-nights per village per month. A trap was hung next to occupants’ foot sleeping under untreated mosquito net about one metre above the ground [23,31] and the trapped female anophelines were collected in the morning by mouth aspirator. PSC was made in the morning (7:00 am to 8:30 am) in ten randomly selected houses in each village once every month. Before spraying, occupants and their domestic animals left the house. In addition, utensils used for food, food, drinking water and clothes were taken out of houses, house apertures carefully covered with clothes, and the available floor was entirely covered by 2–3 white plastic sheets (each having area of 4 m × 5 m). Spraying was made by KILIT™ insecticide aerosol (Miswa Chemicals LTD, Caswell Road, Brackmills, Northampton, NN4 7PW England) according to the manufacturer’s instruction and collectors waited outside for about 15 min. The sheet was then carefully taken out of the house and knocked down mosquitoes were collected using forceps. Five pit traps, constructed in shaded areas, were used for outdoor resting mosquito collection in each village. Each pit shelter was 1.5 m deep, 1.2 m long and 1 m wid. In each pit, four small horizontal cavities of 0.3 m deep were dug out from 0.5 m above the bottom on the walls. Anophelines resting in pit shelters were collected by mouth held aspirator using torch as light source.

Female anophelines from all catches were counted, their abdominal status determined [fresh fed (FF), gravid (GR) or unfed (UF)] and identified morphologically to species under stereoscopic dissecting microscope [23,32]. Unfed anophelines were dissected and their parity determined microscopically as either parous or nulliparous based on changes in their ovarian tracheal system [23]. Each mosquito was kept in a labelled 1.5 ml Eppendorf tube containing silica gel desiccant and cotton. Samples were stored at room temperature while in the field and in -20°C refrigerator at the main laboratory in Addis Ababa until used. FF anophelines were used for blood meal source identification while those of GRs and parous females were used for sporozoite rate determination.

### Identification of *Anopheles gambiae* sibling species by polymerase chain reaction (PCR)

About 12.5% of the *Anopheles gambiae* s.l were selected randomly and identified to their sibling species using species specific polymerase chain reaction (PCR) [33]. A leg was removed from each mosquito and mixed with 12.5 μl PCR master mix (containing 10x dNTPs, MgCl_2_ Solution, QD primer, UN Primer, GA primer, ME primer, AR primer, deionized water and RTag) in 0.2 ml PCR tube, centrifuged for 20s-20min at 16 K r.p.m. and amplified in a PCR apparatus (PTC-100™ Programmable Thermo cycler, MJ Research, Inc., USA) with PCR cycle condition (95°C/5 min × 1 cycle; [95°C/30s, 50°C/30s,72°C/30s] × 30 cycles; 72°C/5 min × 1 cycle; 4°C hold). 5 μl PCR product loaded with 2 μl loading dye and 4 μl DNA ladder were electrophoresed through a 2% agarose-tris-borate-EDTA containing ethidium bromide gel (with 100 V and 150 mA power source) and visualized under UV light box (Alpha Innotech, MultiImage™, Light Cabinet, Pacific Image Electronics Co. Ltd, Taiwan).

### Blood meal source identification and human blood index determination

FF anophelines, from all catches, were assayed for human and bovine blood antigens simultaneously by ELISA [19]. Abdomen of each FF mosquito was ground in 50 μL phosphate-buffered saline (PBS) and final volume brought to 200 μL with PBS buffer. 50 μL of the triturate was coated in duplicate wells on two separate U-bottomed 96-well microtitre plates simultaneously: one plate for human blood meal identification and the other for bovine. Plates were incubated overnight at room temperature and washed twice with PBS-Tween 20. 50 μL peroxidase-conjugated anti-human IgG was added in the first plate and the same volume of peroxidase-conjugated anti-bovine IgG in the second plate incubated for one hour at room temperature and washed thrice with PBS-Tween 20. Finally 100 μL ABTS peroxidase substrate was added, incubated at room temperature for 30 min and observed for green colour reaction visually and absorbance read at 405 nm (by MRX Microplate Reader, Dynex Technologies, 14340 Sullyfield Circle, Chantilly, VA. 20151–1683, USA). Positive control (either human or bovine blood meal) and negative controls (abdomen of laboratory-bred UF *An. arabiensis*) were included in each plate. Human blood index (HBI) and bovine blood index (BBI) of each anopheline species was determined by dividing human fed and cattle fed anophelines respectively to the total tested [13].

### Sporozoite rate (SR) and entomological inoculation rate (EIR) determination

Dried head and thorax of GR or parous mosquito, from all catches, were carefully separated from the abdomen and tested for *P. falciparum* and *P. vivax* circumsporozoite proteins (CSPs) simultaneously [34,35]. Three U-bottomed 96-well micro titre plates were coated separately with 50 μL solution of *P. falciparum*, *P. vivax*-210 and *P. vivax*-247 monoclonal antibodies (MAB) respectively and incubated at room temperature overnight. Contents of plates were drained, washed three times with PBS-Tween 20, filled with 200 μL blocking buffer (BB) and incubated for one hour at room temperature. During the incubation period, mosquitoes were grounded individually in 50 μL boiled casein containing Igepal CA-630 and the final volume brought to 250 μL with BB. BB was removed from plates and 50 μL of each mosquito triturate was added to each of the three test wells. CSP positive sample and laboratory-bred *An. arabiensis* were used as positive and negative controls, respectively. Plates were incubated for two hours and washed with PBS-Tween 20 twice. 50 μL aliquots of homologous peroxidase-conjugated MAB (0.05 μg/50 μL BB) were added to each triplicate well in the plates and incubated for one hour. Plates were washed thrice with PBS-Tween 20, 100 μL ABTS peroxidase substrate added per well and incubated for 30 and or 60 min. Plates were observed visually for green colour and also their optical density determined at 405 nm in the micro plate reader. Samples with green colour and with optical density values of greater than two times the average optical density of the negative controls were considered sporozoite positive. Positive samples were retested for confirmation. The *P. falciparum* and *P. vivax* SRs of each *Anopheles* species was determined by dividing *P. falciparum* and *P. vivax* positive anophelines respectively to the total tested. SR was determined for CDC light trap catches and also for PSCs separately.

Since no human landing catch (HLC) was performed, the daily EIR was estimated based on CDC light trap and PSC. For CDC based EIR, the factor determined for *An. arabiensis* in Zambia [22], where a CDC represents 1.91 of an HLC indoors was used. Thus, 1.91 × (no. sporozoite positive ELISAs/ no. mosquitoes tested) × (no. mosquitoes collected by CDC/no. CDC catches).

Similarly, the daily EIR based on PSC was calculated according to WHO [36] as (no. FF mosquitoes caught by PSC/no. human occupants who spent the night in the sprayed house) × (no. uman fed mosquitoes/no. mosquitoes tested for human blood meal) × (no. sporozoite positive ELISAs/no. mosquitoes tested).

### Statistical analysis

Data entry and analysis was made using SPSS version 16.0 soft ware (SPSS Inc., Chicago, IL). The significance of differences between proportions of human fed and bovine fed anophelines was analysed using Chi-square test. The daily EIR was multiplied by the number of days of the corresponding month to get estimated monthly EIR in each village. Then, the monthly EIRs in each village were summed up to obtain the annual EIR [16].

### Ethical issues

The investigation was ethically approved by the Ethical Committee of the Faculty of Medicine of Addis Ababa University and The National Health Research Ethics Review Committee (NERC) of Ethiopia with reference number RDHE/48-85/2009.

## Results

### Composition and blood meal source of *Anopheles* species

A total of 4558 adult female *Anopheles* mosquitoes were caught of which *Anopheles gambiae* s.l (=*An. arabiensis*) was the most prevalent (53.3%) followed by *Anopheles demeilloni* (26.3%)*, Anopheles christyi* (8.9%), *Anopheles pharoensis* (7.9%) and *Anopheles cinereus* (3.6%) (Table 1). PCR identification of the sample of *An. gambiae* s.l (n=305) showed all to be *An. arabiensis*; hence all other *An. gambiae* s.l samples were regarded to be *An. arabiensis. Anopheles arabiensis* was highest in the low altitude village (86.0%) and lowest in the high altitude village (1.2%). Similarly, *An. pharoensis* was highest in the low altitude village (13.4%) and lowest in the high altitude village (0.3%). On the other hand, catches of *An. christyi*, *An. demeilloni* and *An. cinereus* were highest in the high altitude village and very low or scarce in the low altitude village.

**Table 1:** Anopheline species and their abdominal status by village and collection method in south-central Ethiopia, July 2008- June 2010

**Village**	**Species**	**Total**	** CDC**			** PSC**			** Pit shelter**		
			**UF**	**FF**	**GR**	**UF**	**FF**	**GR**	**UF**	**FF**	**GR**
Hobe (n=2442)	*An. arabiensis*	2101	127	436	272	49	771	427	10	3	6
	*An. pharoensis*	328	34	212	64	0	16	2	0	0	0
	*An. christyi*	7	0	2	5	0	0	0	0	0	0
	*An. cinereus*	5	0	2	1	0	1	1	0	0	0
	*An. demeilloni*	1	0	0	1	0	0	0	0	0	0
Dirama (n=481)	*An. arabiensis*	311	22	65	41	6	118	59	0	0	0
	*An. pharoensis*	26	0	14	3	1	7	1	0	0	0
	*An. christyi*	26	5	12	5	0	0	3	0	1	0
	*An. cinereus*	23	4	12	1	1	3	2	0	0	0
	*An. demeilloni*	95	22	53	14	1	2	1	0	0	2
Wurib (n=1635)	*An. arabiensis*	19	3	6	5	0	4	1	0	0	0
	*An. pharoensis*	5	0	4	1	0	0	0	0	0	0
	*An. christyi*	373	91	149	54	2	45	20	3	2	7
	*An. cinereus*	135	14	56	25	3	13	8	2	2	12
	*An. demeilloni*	1103	128	588	117	9	90	33	22	30	86
	**Total**	**4558**	**450**	**1611**	**609**	**72**	**1070**	**558**	**37**	**38**	**113**

Note: *n*= total anophelines collected per village; *CDC*=Centers for Disease Control light trap; *PSCs*= pyrethriod spray catches; *UF*=Unfed; *FF*=Fresh Fed; *GR*=Gravid.

In almost all species and villages (Table 1), FF anophelines were predominant indoors (in CDC and PSC collections) despite the use of nets by the occupants, whereas these were very low outdoors (in pit shelter collections). Furthermore, UF females of *An. arabiensis* and *An. pharoensis* were surprisingly the lowest indoors in CDC collections. Likewise, significant number of GR females was collected indoors.

Table 2 reveals the blood meal sources of different anopheline species in south-central Ethiopia. In CDC traps, *An. arabiensis* had human blood index (HBI) ranging from 32% in the low altitude village to 57% in the high altitude village (average HBI=34%). In PSC, the same species had HBI of 25% in the high altitude village to 31.5% in the low altitude village (average HBI=31%). In outdoors, very small number of FF *An. arabiensis* were caught and tested from the low altitude village only, which had 66.7% HBI. Thus, the overall HBI of *An. arabiensis* in the study area was 32.2%. Regarding *An. pharoensis*, the HBI in CDC traps ranged from 19% in the low altitude village to 21.4% in the mid altitude village (average HBI=18.8), whereas its values in the PSC ranged from 25% in the low to 0% in the mid village (average HBI=17.4%); no specimen was analysed from pit shelters. Thus, the overall HBI of *An. pharoensis* was 18.6% in the study area.

**Table 2:** Blood meal sources of indoor and outdoor resting anophelines of three highland villages (Hobe, Dirama and Wurib) of south-central Ethiopia, July 2008- June 2010

**Village and anopheline**	** CDC**					** PSC**					** Pit shelter**				
	**n**	**HBI**	**BBI**	**Mix**	**Un**	**n**	**HBI**	**BBI**	**Mix**	**Un**	**n**	**HBI**	**BBI**	**Mix**	**Un**
**Hobe**															
*An. arabiensis*	422	32	39.1	13.7	15.2	723	31.5	39.4	12.2	16.9	3	66.7	33.3	0	0
*An. pharoensis*	206	18.9	55.8	14.6	11.6	16	25	43.8	25	6.2	0	0	0	0	0
**Dirama**															
*An. arabiensis*	64	43.7	34.4	12.5	9.4	114	28.1	49.1	7	15.8	0	0	0	0	0
*An. pharoensis*	14	21.4	64.3	14.3	0	7	0	85.7	14.3	0	0	0	0	0	0
*An. christyi*	9	11.1	66.7	11.1	11.1	1	0	100	0	0	1	100	0	0	0
*An. cinereus*	10	20	60	20	0	2	50	50	0	0	0	0	0	0	0
*An. demeilloni*	41	9.8	70.7	2.4	17.1	1	0	0	0	0	0	0	0	0	0
**Wurib**															
*An. arabiensis*	6	57.1	14.3	0	28.6	4	25	0	25	50	0	0	0	0	0
*An. christyi*	125	26.4	55.2	8	10.4	37	27	48.6	18.8	5.6	2	50	50	0	0
*An. cinereus*	49	20.4	51	14.3	14.3	12	16.7	66.7	8.3	8.3	1	100	0	0	0
*An. demeilloni*	471	11.5	69	5.7	13.8	70	5.7	72.9	1.4	20	22	9.1	68.2	0	22.7

Note: *n*= number tested; *HBI*=human blood index in%; *BBI*=bovine blood index in%; *Mix*= human and bovine mixed blood index (%); *Un*=unidentified blood meal in%.

Regarding the zoophilic feeding behaviour of the two species, *An. arabiensis* had bovine blood feeds of 14.3% in the high elevation village to 39.1% in the low (average=38%) in CDC catches, while the values ranged from 0% in the high altitude village to 49% in the mid-altitude village (average=40.5%) in PSCs. In outdoor catches, only 33.3% were bovine fed. Thus, the overall zoophilic feeding pattern (index) was about 39.6% in the study area. For *An. arabiensis*, its overall BBI was not statistically different from the HBI.

Similarly, *An. pharoensis* which was absent in the high altitude village had 55.8% and 64.3% of similar bovine feeding rates in the low and mid villages in CDC catches, respectively (average=51.2%). It also had BBIs of 43.8% in the low and 85.7% in the mid village (average=56.5%). In the absence of bovine feeds outdoors, its overall bovine feeding rate was 55.9% showing to have a more zoophilic behaviour than that of *An. arabiensis.* However, the overall BBI of *An. arabiensis* was not significantly different from that of the BBI of *An. pharoensis*.

Apart from either of the two main blood meal sources (human and bovine), a small proportions of the two species also had mixed feeding patterns ranging from 0 to 14% in CDC traps and from 7 to 25% in PSCs with averages of 12.2% for *An. arabiensis* and 15% for *An. pharoensis*. Furthermore, 15.2% *An. arabiensis* and 11.6% of *An. pharoensis* from Hobe had blood meals of undetermined origin; no such blood meals were detected in outdoor pit shelters since specimens were generally low. Other non-vector anophelines (*An. christyi*, *An. cinereus* and *An. demeilloni*) caught indoors or outdoors in all villages exhibited far more zoophilic behaviour (48.6% to 100%) than anthropophilic behaviours.

### Sporozoite rates

A total of 1117 indoor caught anophelines, representing five species, were tested for *Plasmodium* circumsporozoite proteins (CSPs) (Table 3). Sporozoites were only detected in 18 mosquitoes belonging to two species (*An. arabiensis* and *An. pharoensis*) collected from the low and mid-altitude villages. A total of 819 *An. arabiensis* tested from both CDC and PSC had overall *P. vivax* and *P. falciparum* sporozoite rates of 1.7% and 0.2%, respectively. In the low altitude village, the *P. vivax* sporozoite rate in the same species was 3% and 0.7% from CDC and PSC, respectively, where highest number of *An. arabiensis* was caught and analysed. The *P. falciparum* sporozoite rate for the same mosquito in the village was 0.3% and 0.2% in the CDC and PSC, respectively. In the mid altitude village, where small number of *An. arabiensis* were analysed, the *P. vivax* rates were 2.5% (1/40) (CDC) and 1.7% (1/58) (PSC).

**Table 3:** Sporozoite infection rates of anophelines in three highland villages of south-central Ethiopia, July 2008- June 2010

**Villages and parameters**	***An. arabiensis***		***An. pharoensis***		***An. demeilloni***		***An. christyi***		***An. cinereus***		**Total**
	**CDC**	**PSC**	**CDC**	**PSC**	**CDC**	**PSC**	**CDC**	**PSC**	**CDC**	**PSC**	
**Hobe**											
No. tested	300	416	71	2	0	0	1	0	1	0	791
No. PvS+ (%)	9 (3)	3 (0.7)	1 (1.4)	1 (50)	0	0	0	0	0	0	14 (1.8)
No. PfS+ (%)	1 (0.3)	1 (0.2)	0	0	0	0	0	0	0	0	2 (0.3)
**Dirama**											
No. tested	40	58	4	1	12	0	3	2	1	3	124
No. PvS+ (%)	1 (2.5)	1 (1.7)	0	0	0	0	0	0	0	0	2 (1.6)
No. PfS+ (%)	0	0	0	0	0	0	0	0	0	0	0
**Wurib**											
No. tested	4	1	1	0	85	22	45	10	28	6	202
No. PvS+ (%)	0	0	0	0	0	0	0	0	0	0	0
No. PfS+ (%)	0	0	0	0	0	0	0	0	0	0	0
**Total**											
No. tested	344	475	76	3	97	22	49	12	30	9	1117
No. PvS+ (%)	10 (2.9)	4 (0.8)	1 (1.3)	1 (33)	0	0	0	0	0	0	16 (1.4)
No. PfS+ (%)	1 (0.3)	1 (0.2)	0	0	0	0	0	0	0	0	2 (0.3)
**Overall**											
No. tested	819		79		119		61		39		1117
No. PvS+ (%)	14 (1.7)		2 (2.5)		0		0		0		16 (1.4)
No. PfS+ (%)	2 (0.2)		0		0		0		0		2 (0.3)

*PvS+ (%)*= number *P. vivax* sporozoite positive (rate in percent) ; *PfSR (%)* = number *P. falciparum* sporozoite positive (rate in percent).

Similarly, analysis of only 79 *An. pharoensis* from all the three villages resulted in an overall *P. vivax* rate of 2.5% (2/79) with no *P. falciparum* infection. Most of the *An. pharoensis* caught and analysed was from the low altitude village where *P. vivax* sporozoite rate was 1.4% (1/71) in CDC and 50% (1/2) in PSC. None of the very few mosquitoes tested in the two other villages were positive for either of the two *Plasmodium* sporozoites.

Although sporozoite infections were generally low, they were higher among CDC light trap catches (Table 4) than PSC catches (Table 5). Among the *An. arabiensis* caught by the CDC trap, the daily *P. vivax* sporozoite rate was highest in May 2010 (which was 0.2) and was lower or zero during most of the months in the low altitude village (Table 4) where most of the sporozoites were observed. No distinct seasonal pattern was apparent for *An. pharoensis* since only two mosquitoes were found positive for *P. vivax* during the whole study period. Generally, very low *P. falciparum* sporozoite rate were observed in all catches and study villages.

**Table 4:** CDC light trap based assessment of sporozoite and entomological inoculation rates in two highland villages of south-central Ethiopia, July 2008- June 2010

**Study period**	**Hobe**				**Hobe**		**Dirama**	
	***An. arabiensis***				***An. pharoensis***		***An. arabiensis***	
	**Daily PvSR**	**Monthly PvEIR**	**Daily PfSR**	**Monthly PfEIR**	**Daily PvSR**	**Monthly PvEIR**	**Daily PvSR**	**Monthly PvEIR**
Jul 2008	0	0	0	0	0	0	0	0
Aug 2008	0.17	6.23	0	0	0	0	0	0
Sep 2008	0	0	0	0	0	0	0	0
Oct 2008	0.04	4.00	0	0	0	0	0	0
Nov 2008	0	0	0	0	0	0	0	0
Dec 2008	0	0	0	0	0	0	0	0
Jan 2009	0	0	0	0	0	0	0	0
Feb 2009	0	0	0	0	0	0	0	0
Mar 2009	0.05	3.85	0	0	0	0	0	0
Apr 2009	0.03	11.56	0	0	0	0	0	0
May 2009	0.03	7.31	0.02	3.66	0	0	0	0
Jun 2009	0	0	0	0	0	0	0	0
**Year I Total**	**0.32**	32.95^*^	**0.02**	3.66^*^	**0**	0^*^	**0**	0^*^
Jul 2009	0	0	0	0	1	2.3	0	0
Aug 2009	0	0	0	0	0	0	0.5	2.58
Sep 2009	0	0	0	0	0	0	0	0
Oct 2009	0	0	0	0	0	0	0	0
Nov 2009	0	0	0	0	0	0	0	0
Dec 2009	0	0	0	0	0	0	0	0
Jan 2010	0	0	0	0	0	0	0	0
Feb 2010	0	0	0	0	0	0	0	0
Mar 2010	0	0	0	0	0	0	0	0
Apr 2010	0	0	0	0	0	0	0	0
May 2010	0.2	14.5	0	0	0	0	0	0
Jun 2010	0	0	0	0	0	0	0	0
**Year II Total**	**0.2**	14.5^*^	**0**	0^*^	**1**	2.3^*^	**0.5**	2.58^*^

*PvEIR*=*Plasmodium vivax* entomological inoculation rate; *PfEIR*=*P. falciparum* entomological inoculation rate; ^*^= annual EIR.

**Table 5:** PSC based assessment of sporozoite and entomological inoculation rates in two highland villages of south-central Ethiopia, July 2008- June 2010

	**Hobe**				**Hobe**		**Dirama**	
	***An. arabiensis***				***An. pharoensis***		***An. arabiensis***	
**Study period**	**Daily PvSR**	**Monthly PvEIR**	**Daily PfSR**	**Monthly PfEIR**	**Daily PvSR**	**Monthly PvEIR**	**Daily PvSR**	**Monthly EIR**
Jul 2008	0	0	0	0	0	0	0	0
Aug 2008	0	0	0	0	0	0	0	0
Sep 2008	0	0	0	0	0.5	0.32	0	0
Oct 2008	0.08	0.13	0	0	0	0	0	0
Nov 2008	0	0	0	0	0	0	0	0
Dec 2008	0	0	0	0	0	0	0	0
Jan 2009	0	0	0	0	0	0	0	0
Feb 2009	0	0	0	0	0	0	0	0
Mar 2009	0	0	0	0	0	0	0	0
Apr 2009	0	0	0	0	0	0	0	0
May 2009	0	0	0	0	0	0	0	0
Jun 2009	0.03	0.73	0	0	0	0	0	0
**year I Total**	**0.11**	0.86^*^	**0**	0^*^	**0.5**	0.32^*^	**0**	0^*^
Jul 2009	0	0	0	0	0	0	0	0
Aug 2009	0	0	0	0	0	0	0	0
Sep 2009	0	0	0.04	0.93	0	0	0	0
Oct 2009	0	0	0	0	0	0	0	0
Nov 2009	0	0	0	0	0	0	0	0
Dec 2009	0	0	0	0	0	0	0	0
Jan 2010	0	0	0	0	0	0	0	0
Feb 2010	0	0	0	0	0	0	0	0
Mar 2010	0	0	0	0	0	0	0	0
Apr 2010	0	0	0	0	0	0	0	0
May 2010	0	0	0	0	0	0	0.09	0.2
Jun 2010	0	0	0	0	0	0	0	0
**Year II Total**	**0**	0^*^	**0.04**	0.93^*^	**0**	0^*^	**0.09**	0.2^*^

### Entomological inoculation rates (EIR)

In the absence of direct human landing catches, EIR for each village was estimated based on the sampling methods employed (CDC and PSCs). However, a small number of mosquitoes were found sporozoite positive on both catches and in all villages. This resulted in low EIR estimates varying from 0 (in most months) to 14.5 (May 2010) monthly *P. vivax* infectious bites of *An. arabiensis* in the low altitude village (Hobe) based on the CDC trap catches (Table 4), while it had only 2.58 in the mid-altitude village (Dirama) in August 2009. Based on CDC based EIR estimates, there was evidence of *P. vivax* transmission in August and October of 2008, in March, April and May of 2009, and in May 2010 coinciding with small rainy seasons of the year in the area.

Although the number of *An. arabiensis* caught by PSC (n=1,247) was much higher than the number caught by CDC traps (n=835), in the low altitude village, the total number of sporozoite infected mosquitoes was very low in the PSC (Table 5). Monthly *P. vivax* EIRs of 0.13 and 0.73 were observed in October 2008 and in June 2009 in the village. In addition, there was *P. falciparum* EIR of 0.93 in September 2009 in the village. While the only *P. vivax* infection in the mid-altitude village in May 2010, resulted in the monthly EIR of 0.2.

Annual EIRs varied between the first and the second years and also between the low and mid-altitude villages (Table 4). From the CDC light trap collections; there were 3.66 and 0 *P. falciparum* infective bites per year per person for *An. arabiensis* in the years July 2008 to June 2009 and July 2009 to June 2010 respectively in the low altitude village. The corresponding values for *P. vivax* infective bites by *An. arabiensis* were 33 and 14.5 in the village. In addition, there were 0 and 2.3 *P. vivax* infective bites for *An. pharoensis* in the village during the first year and the second year, respectively. The space spray catch revealed 0.32 *P. vivax* infective bites per person for *An. pharoensis* during the first year with zero value in the second year.

## Discussion

*Anopheles arabiensis* was the predominant malaria vector followed by *An. pharoensis* along the altitudinal transect consisting of Hobe (low altitude; 1,800 m), Dirama (mid altitude; 2,000 m) and Wurib (high altitude; 2,200 m) villages in south-central Ethiopia. Although sampling was not made for anophelines that could escape through eves and windows, the highest number of *An. arabiensis* was caught by pyrethroid spray revealing its indoor resting behaviour [37,38]. The majority of the anophelines were collected from inside houses which could be associated with the indoor occurrence of blood meal sources, higher indoor temperature and with limited outdoor-resting places [38,39]. Most *An. arabiensis* and other anopheline species caught indoors (*An. pharoensis*, *An. christyi*, *An. demeilloni*, and *An. cinereus*) were fresh fed and gravid indicating their indoor or outdoor feeding with indoor resting behaviour. The higher number of fresh fed and gravid mosquitoes in the CDC light trap catches might be due to their attraction to CDC light traps and their possible repeated feeding behaviour [13,22,27]. The human fed catches by the CDC light traps, despite the presence of nets, might be due to the early biting behaviour of *An. arabiensis*[40] before bed time and blood feeding on exposed occupants who sleep traditionally on floor mats in which case nets do not provide adequate protection.

The HBI of *An. arabiensis* was similar to that of its BBI indicating its opportunistic feeding behaviour in the area. Similar feeding preferences are reported from southern Ethiopia where people and livestock either share the same houses or where cattle are kept separate but close to houses during the night [41]. Our result can also be strengthened by the study from Fuchucha village in the Konso District of Ethiopia where cattle- and human-fed *An. arabiensis* mosquitoes were found to have similar rates of *Plasmodium* infection [42]. However, the HBI observed in this study is very low compared to the value from human dwellings alone (91.5%) and higher compared to that from human and bovine mixed dwellings (20.2%) reported in the country [43]. The variations in the HBI of the vector could result from differences in the relative distance and accessibility of hosts.

The HBI of *An. pharoensis* observed in this study (18.6%) is lower than that of *An. arabiensis,* but is higher compared to that of the Kenya (8.2%) [13]. In addition, it had the highest mixed human and bovine blood index among the five anopheline species. An experimental study in southern Ethiopia [44] documented similar number of *An. pharoensis* catches both in human- and cattle-baited traps. Thus, it can be suggested that *An. pharoensis* of south-central Ethiopia may have a moderately opportunistic feeding behaviour probably due to its similar responsiveness to cattle and human host cues [44]. This tendency of the mosquito to feed on humans increases its vectorial capacity. *Anopheles christyi, An. cinereus* and *An. demeilloni* also had considerably high human blood indices depicting their importance as biting nuisances. *Anopheles cinereus* has previously been reported as a potential vector of malaria in Eritrea [45]. Significant number of blood meals of *An. arabiensis*, *An. pharoensis* and other anophelines could not be identified by ELISA, which most could have been identified by PCR [33]. Limitations of primers and reagents hindered the use of such a technique in the study. The quality of some of the blood samples might have also been degraded during storage before analysis. However, the unidentified blood meal sources could be of sheep, goat, donkey, horse, chicken and dogs which are available in the area.

*Anopheles arabiensis* was the most abundant, most anthropophilic and the most sporozoite laden species proving its role as the primary malaria vector in the area [1]. Very few *An. pharoensis* (n=2) were found carrying *P. vivax* sporozoites which might be attributed to its occurrence mainly following the main rainy season. *P. vivax* sporozoite carriage was higher than that of *P. falciparum* which is also similar to previous reports from southern Ethiopia [41,42,46]. This describes dominance of vivax malaria transmission over falciparum in the region. It is, therefore, imperative to undertake epidemiological studies on *P. vivax* in view of the current reports that revealed severe clinical manifestation resulting from the infection [47,48].

Annual *Plasmodium falciparum* infectious bite was lower than 10 in the study villages indicating its unstable transmission intensity [21,49] and risk of epidemics [50]. Apart from this, the study area is a highland fringe where vector density is lower resulting in low transmission intensity compared to typical lowland malarious areas such as in southern Ethiopia [42], Tanzania [51], Eritrea [14], Zambia [16] and Uganda [49]. For example, in southern Ethiopia, more than 45, 000 *An. arabiensis* were collected in 12 months at a locality with an average altitude ranging from 800 m to 1,300 m a.s.l. [42] compared to the present 2,431 *An. arabiensis* in the two years study time. However, since adult anopheline sampling was undertaken only once per month, this value may underestimate the risk of malaria transmission. *Plasmodium falciparum* and *P. vivax* infective *An. arabiensis* bites and *P. vivax* infective *An. pharoensis* bites decreased starting from the low altitude village to the higher. An increase in altitude is related to a decrease in temperature that limits vector occurrence and development of the parasites in the vector thereby reducing the number of infectious anopheline bites [50,52,53]. Mortality due to malaria was also reported to have a decreasing magnitude with increasing altitude in the area [28].

Recent studies, in the study area [7,54], reported *P. vivax* and *P. falciparum* malaria transmissions at elevations ranging from 2,100 m to 2,280 m. Although the relationship between EIR and malaria prevalence rate is not direct [20,55], EIR may vary between 0 and 1,500 infective bites per person per year in endemic countries of Africa and is a useful index in assessing malaria endemicity and transmission intensity [20,49]. The number of infective bites by both *An. arabiensis* and *An. pharoensis* were higher during dry months compared to the rainy months as was observed in western Kenya [56] and eastern Sudan [57]. However, this trend is different from the report in Eritrea [14] where EIR in wet season was nine times higher than in the dry season and also from that of southern Zambia [16], Tanzania [51] and Kenya [58]. This seasonal difference could result from diverse ecological adaptation and behavioural changes of *An. arabiensis*. Malaria infectious bites were observed during the months of August to October and March to June which generally corresponds to the major and minor malaria transmission seasons respectively in the country [59]. This finding suggests that malaria transmission is seasonal and unstable in Hobe, Dirama and Wurib villages of south-central Ethiopia. As in most parts of Ethiopia, the unstable malaria transmission in the study area could result from variations in the meteorological factors, movement of inhabitants from non malarious to malarious areas and vice versa, and human population growth increasing activities that create increased and suitable vector breeding habitats along natural wetlands and foothills [60]. In addition, *Plasmodium* infectious bites were more frequent in the first study year (July 2008 to June 2009) and decreased from the low elevation village to the suggesting temporal and spatial variation of malaria transmission intensity.

## Conclusion

*Anopheles arabiensis* was the most prevalent vector of *P. vivax* and *P. falciparum* malaria along a south-central highland transect of Ethiopia consisting of Hobe (low altitude), Dirama (mid altitude) and Wurib (high altitude) villages followed by *An. pharoensis*. Both anopheline species fed on human and bovine of which the first was opportunistic feeder while the second being moderately anthropophilic. The annual EIRs were generally lower compared to typical endemic areas and showed a decreasing trend from the low altitude village to the high altitude village.

## Competing interests

The authors declare that they have no competing interests.

## Authors’ contributions

AA designed the study, collected data in the field, carried out the data analysis and wrote the first draft of the manuscript. TGM participated in the study design, interpretation of the results and editing the manuscript. MB participated in the conception of the study, in the study design and editing the manuscript. BL conceived the idea for the study and took part in the study design, data entry and analysis, data interpretation and editing the manuscript. All authors have read and approved the final manuscript.
